# Structural requirements for the binding affinity of some small, non–peptide C5a receptor antagonists

**DOI:** 10.1038/npre.2011.6630.1

**Published:** 2011-11-21

**Authors:** Haiying Xie, Weiming Wang, Ziqiang Xie, Hong Chen, Kexian Chen

**Affiliations:** 1 Zhejiang Xinhua Chemical Co., Ltd, Jiande, Zhejiang 311600 China; 2 Zhejiang University, Department of Chemistry, Hangzhou, Zhejiang 310027 China

**Keywords:** C5a receptor, Antagonist, inflammation, Quantitative structure–activity relationship, Structural requirements

## Abstract

Complement anaphylatoxin 5a (C5a) has been recognized as a potent therapeutic target for anti-inflammatory therapy, thus, blocking the action of C5a on its binding receptors may provide an effective treatment of a variety of inflammatory diseases. However, there have been few clinically available non-peptide C5a receptor antagonists disclosed at present. In pursuit of better anti-inflammatory drugs, quantitative structure–activity relationship studies were carried out in a series of non-peptide C5a receptor antagonists with binding activity using different physicochemical descriptors. The conventional best 2D-QSAR models were developed using a training set of 35 molecules and an external test set of 8 molecules by genetic function approximation (GFA) and stepwise multiple linear regression (Stepwise-MLR) with acceptable r^2^ of 0.773 and 0.863, r^2^~CV~ of 0.752 and 0.775, and r^2^~pred~ of 0.801 and 0.888, respectively, indicating binding activity strongly depends on thermodynamic properties as expressed by the hydrophobicity of molecules.

### Introduction

Prolonged activation of the host defense human complement system of plasma proteins contributes significantly to amplifying the inflammatory and cellular responses to stimuli such as infectious microorganisms, chemical and physical injury, radiation, or neoplasia (Lama *et al.*, 1992; Finch *et al.*, 1999), resulting in a cascade of proteolytic cleavages of complement proteins Cl-C5 (Vlattas *et al.*, 1994; Wong *et al.*, 1998), assembly of the membrane attack complex capable of cell lysis (Lama *et al.*, 1992) and release of numerous complement-derived peptides of the anaphylatoxins of C3a, C4a and C5a that interact with cellular components to propagate the inflammatory process (Lama *et al.*, 1992; Vlattas *et al.*, 1994). Biological activities of these anaphylatoxins (Lama *et al.*, 1992) include the cellular release of vasoactive amines and lysosomal enzymes, contraction of smooth muscle, and enhanced vascular permeability. Although broad features of complement activation are known, the details of pathogenesis remain largely unknown (Wong *et al.*, 1998; Harkin *et al.*, 2004).

As is shown in literatures, C5a, a 74-amino acid peptide cleaved from C5 at sites of inflammation or infection during activation of the complement system (CS) (Blagg *et al.*, 2008a, 2008b), is a broad pro-inflammatory molecule that binds to G protein-coupled receptors CD88 (C5aR) (Schnatbaum *et al.*, 2006; Barbay *et al.*, 2008). It has been recognized as a very potent inflammatory mediator generated during complement activation (Wong *et al.*, 1998) and a causative or aggravating agent in a variety of inflammatory diseases including rheumatoid arthritis, inflammatory bowel disease, immune complex disease, reperfusion injury, Alzheimer's disease, ischemic heart disease, and adult respiratory distress syndrome (ARDS) (Vlattas *et al.*, 1994; Wong *et al.*, 1998; Finch *et al.*, 1999; Haas *et al.*, 2005). It possesses additional chemotactic biological activities (Lama et al., 1992; Vlattas *et al.*, 1994) that are mediated through specific receptor-ligand interactions, including an increase in $Ca^{2+}$ mobilization, activation of neutrophil chemotaxis and aggregation, stimulation of leukotriene and oxidative product release, induction of interleukin-1 transcription by macrophages, enhanced antibody production, and other strong pro-inflammatory response. Thus, C5a is a very intriguing therapeutic target for anti-inflammatory therapy (Haas *et al.*, 2005), blocking the action of C5a on its binding receptors may provide an effective treatment of a variety of inflammatory diseases (Lama *et al.*, 1992; Arumugam *et al.*, 2004, Blagg *et al.*, 2008a, 2008b). Considerable efforts have also been directed toward the discovery of small molecule drugs capable of blocking the complement C5aR response especially, but there have been few clinically available non-peptide C5a receptor antagonists disclosed (Astles *et al.*, 1997; Schnatbaum *et al.*, 2006). For these reasons, it is necessary and also urgent to further understand the C5a structural features important for receptor binding and activation (Vlattas *et al.*, 1994; Finch *et al.*, 1997).

Computational chemistry has been applied widely in the pharmaceutical industry for drug discovery, lead optimization, risk assessment, toxicity prediction and regulatory decisions

Nature Precedings : doi:10.1038/npre.2011.6630.1 : Posted 19 Nov 2011

(Sharma *et al.*, 2008). Traditional computer-assisted quantitative structure–activity relationship (QSAR) studies pioneered by Hansch *et al.* (1962) provide a rational basis to establish the relationship between physiochemical properties and biological activity of molecules for better understanding the mechanisms of biological performance and show how to improve performance by altering chemical structures of ligands, which increase the probability of success and reduce the time and cost involved in the modern drug discovery process (Neaz *et al.*, 2008). Besides, QSAR method save resources and expedite the process of the development of new molecules and drugs. There have been many QSAR researches related to modern drug design since it was first introduced. The aim of present work is to derive statistically some significant quantitative structure- activity relationship (QSAR) models for structural requirements of the binding affinity of some non-peptide C5a receptor antagonists, which would aid in search for the novel orally available non-peptide C5a receptor antagonists prior to synthesis.

Nature Precedings : doi:10.1038/npre.2011.6630.1 : Posted 19 Nov 2011

### Materials and Methods

#### Data set

A data set of some small, non–peptide C5a receptor antagonists were taken from the published work (Blagg *et al.*, 2008a, 2008b). Their C5a receptor binding activity data [$^{125}$Binding affinity $IC_{50}$ (nM)] were taken in molar (M) range and then converted into the corresponding logarithmic values ($pIC_{50}$) according to the formula: $pIC_{50}$ = -log$IC_{50}$. Out of reported 54 molecules, 11 molecules were discarded for which the precise data were not available. The remaining molecules (Table 1) were manually segregated into training (35 molecules) and test (8 molecules) sets (Table 2) based on the suggestions by Oprea *et al.* (1994), maintaining the structural diversity and wide range of activity in both sets for the subsequent QSAR analysis.

**[Insert Table 1 and Table 2]**

**Model building**

All computational experiments were performed using Cerius$^2$ (version 4.10) running on Silicon Graphics O2 R5000 workstation. The molecular geometric structures were constructed using a 3D-sketcher in the Cerius$^2$ Builder option and partial charges assigned using the Gasteiger method (Gasteiger and Marsili, 1980). Throughout the study, an energy minimization procedure named universal force field 1.02 (Rappe *et al.*, 1992) was employed to generate the lower energy conformation for each molecule. All the structures were subsequently energy minimized until a root mean square derivation 0.001 kcal/ mol· Å was achieved and used in this study (Deokar *et al.*, 2008).

**Calculation of descriptors**

Different types of physicochemical descriptors for each molecule were generated in the study table using default setting within QSAR+ module of Cerius$^2$. There were total 242 nonzero descriptors including E-state-indices, Information_content, conformational, thermodynamic, topological, electronic, structural, and spatial descriptors. Before generating models, the inter-correlation of descriptors was considered and the descriptors with value over 0.7 were removed (Shen *et al.*, 2004). Descriptors used for model generation are listed and described in Table 3.

**[Insert Table 3]**

### Genetic function approximation (GFA)

In the present study, genetic function approximation (GFA) was used to generate 2D-QSAR models (Kelkar *et al.*, 2004; Deokar *et al.*, 2008). GFA, developed by Rogers and Hopfinger (1994), was genetically involved in the combination of Fried machs multivariate adaptive regession splines (MARS) (Friedman, 1991) and Holland's genetic algorithem (GA) (Holland, 1975). It is a useful statistical analysis tool to correlate biological activity or property with molecular characteristic parameters, and also greatly improves the ease of successful model interpretation. The length of GFA derived equation was initially fixed to five terms including a constant suggested by Deokar *et al.* (2008). The population size was established as 100, the equation term was set to linear polynomial and the mutation probability was specified as 50%. After some preliminary runs for observations, GFA crossover of 10000 and smoothing parameter "d" value of 2.0 were set to give reasonable convergence. Other settings were maintained at their default values.

### Stepwise multiple linear regression (Stepwise-MLR)

The stepwise multiple linear regression (Stepwise-MLR) procedure was also employed for the model selection on account of many descriptors used in this study. The multiple linear regression method with stepwise selection calculates QSAR equations by adding one variable at a time and testing each addition for significance (Jung *et al.*, 2007). Only variables tested to be significant are finally used in the QSAR equation. This regression method is especially useful when the number of variables is large and the key descriptors for the activity are not known. The forward regression calculation mode was selected in this study because backward regression calculation can lead to overfitting. The maximum number of steps to be run in the calculation was set at 100, which can be specified to avoid hysteresis. F value of 4.000 was to evaluate the significance of a variable when a variable is added to or deleted from the equation. If the F value of a variable falls below a specified value, the variable is removed.

Nature Precedings : doi:10.1038/npre.2011.6630.1 : Posted 19 Nov 2011

Nature Precedings : doi:10.1038/npre.2011.6630.1 : Posted 19 Nov 2011

## Results and discussion

### GFA-derived QSAR model

The number of descriptors necessary and adequate in the GFA-derived QSAR equations was investigated in the beginning. As the conventional square correlation coefficient ($r^2$) can be easily increased by number of terms in the equation, the cross-validated $r^2$ ($r^2_{CV}$) was selected as the limiting factor for the number of descriptors in the equation (Nair and Sobhia, 2008). As shown in Figure 1, the $r^2_{CV}$ value increases till the number of descriptors in the equation reaches up to 4 and $r^2_{CV}$ value starts decreasing as the number of descriptors increases further. Thus, the number of descriptors in the equation was restricted to 4 for the final model. The set of equations generated was evaluated on the following basis:

a. Square correlation coefficient ($r^2$)

b. Friedman's lack of fit score (LOF)

c. Cross-validated $r^2$ ($r^2_{CV}$)

d. External predictive power of the model ($r^2_{pred}$)

The statistically significant GFA-derived QSAR model with four descriptors below is shown:

Model-I

$$pIC_{50} = 1.97432 + 0.413266(Atype\_C\_25) + 0.15099(Atype\_H\_52) + 0.670423(AlogP) - 0.306762(logP)$$

$N_{(Training\ set)} = 35$, LOF = 0.174, $r^2 = 0.773$, $r^2_{adj} = 0.743$, F-text = 25.537, LSE = 0.104, r = 0.879, $r^2_{CV} = 0.752$, $r^2_{BS} = 0.774$, PRESS = 5.442, $N_{(test\ set)} = 8$, $r^2_{pred} = 0.801$

where $N_{(Training\ set)}$ is the number of compounds in training set; LOF is Friedman's lack of fit score (Deokar *et al.*, 2008); $r^2$ is the squared correlation coefficient; $r^2_{adj}$ is square of adjusted correlation coefficient; F-test is the variance related static; LSE is the least square error; r is the correlation coefficient; $r^2_{CV}$ is a squared correlation coefficient generated during the cross-validation procedure; Bootstrap $r^2$ ($r^2_{BS}$) (Deokar *et al.*, 2008) is the average squared correlation coefficient calculated during the validation procedure; PRESS, predicted sum of deviation squares, is the sum of overall compounds of the squared differences between the actual and the predicted values for the dependent variables; $N_{(test\ set)}$ is the number of compounds in test set; $r^2_{pred}$ is the predictive power of the model.

**[Insert Figure 1]**

The inter-correlation of the descriptors appeared in the above best model was taken into account and the descriptors were found to be reasonably orthogonal. Main descriptor values appeared in the above 2D-QSAR model-I of training set and test set molecules are shown in Table

2.

**[Insert Table 4]**

The full cross-validation tests and randomization tests were employed to determine reliability and significance of these generated models. The full cross-validation tests (Fan *et al.*, 2001) encompass the entire algorithm, including both the choice of descriptors and the optimization of regression coefficients. The full cross-validated $r^2$ ($r^2_{CV}$) was computed using the predicted values of the missing molecules by the models obtained from the remaining compounds in the data set. The results based on the rules of “leave-1-out”, “leave-2-out”, “leave-3-out”, “leave-4-out”, “leave-5-out”, “leave-6-out”, “leave-7-out”, “leave-8-out”, “leave-9-out” and “leave-10-out” are shown in Table 4, indicating the results obtained were not by chance correlation. The randomization tests (Deswal and Roy, 2006; Nair and Sobhia, 2008) were performed at 90% (9 trials), 95 % (19 trials), 98 % (49 trials) and 99% (99 trials) confidence levels and carried out by repeatedly permuting the dependent variable set. The results of randomization tests in Table 5 showed that none of the permuted data sets produced the random r comparable to nonrandom r of 0.879, suggesting that the value obtained for the original GFA model was significant. The predictive power of the model-I was also evaluated by the external test set molecules. The predictive power of the model-I was calculated by $r^2_{pred}$ = (SD-PRESS)/SD (Deokar *et al.*, 2008; Deswal and Roy, 2006; Nair and Sobhia, 2008), where SD is the sum of squared deviations between the $pIC_{50}$ of each molecule and the mean $pIC_{50}$ of the molecules in the training set and PRESS is the sum of squared deviations between the predicted $pIC_{50}$ and actual $pIC_{50}$ values for every molecule in the test set. The high $r^2_{pred}$ value of 0.801 for the test set accounted for good predictive ability. The developed QSAR model-I thus was robust and was found satisfactory for predicting the activities of the test set (Table 2). From Table 2, molecule **17** turned out to have high residuals because of their high activities in comparison to other compounds.

**[Insert Table 5 and Table 6]**

According to model-I, the observed C5a receptor binding activity for these non–peptide C5a receptor antagonists are principally influenced by Atype_C_25, Atype_H_52, AlogP and logP, which is confirmed by the maximum frequent usage of these descriptors during the formation of models (Table 6). All of these descriptors in model-I belong to thermodynamic character. LogP is the partition coefficient (Deswal and Roy, 2006), which represents the lipophilicity of molecule. The negative slope of logP in this equation represents that activity decreases with an increase in lipophilicity of molecule, which can be obviously shown in Table 2. Thus, substituents, which increase lipophilicity of compound, should be avoided. Descriptors of Atype_C_25 and Atype_H_52 are the atom type AlogP descriptors used to characterize the hydrophobicity (logP) of molecules. The atomic contribution of individual atom types was proposed by Ghose and

Nature Precedings : doi:10.1038/npre.2011.6630.1 : Posted 19 Nov 2011

Nature Precedings : doi:10.1038/npre.2011.6630.1 : Posted 19 Nov 2011

Crippen (1987) toward the overall hydrophobicity of molecules where carbon, hydrogen, oxygen, nitrogen, sulfur and halogens were classified into 120 atom types (Deswal and Roy, 2006; Nair and Sobhia, 2008). Hydrogen and halogens are classified by the hybridization and oxidation state of the carbon they are bonded to; carbon atoms are classified by their hybridization state and the chemical nature of their neighboring atoms. A total of 44 carbon types alone attest the complexity of the classification procedure. The positive slope of Atype_C_25 and Atype_H_52 in model-I represents that activity increases with an increase in lipophilicity related to C_25 and H_52 atom types for these molecules. The atom type C_25 (Ghose and Crippen, 1987; Nair and Sobhia, 2008) is C in :R--CR--R and Atype_H_52 is H that is unused where R represents any group linked through carbon and -- represents aromatic bonds as in benzene or delocalized bonds as the N-O bond in nitro group. Hydrophobicity associated with C atom as part of the aromatic ring or N-O bonded in nitro group is favorable for C5a receptor binding activity ($pIC_{50}$).

### Stepwise-MLR-derived QSAR model

The stepwise multiple linear regression (Stepwise-MLR) procedure was employed to correlate the variations of biological activities with the various physicochemical properties and select significant model by adding one variable at a time and testing each addition. The activity ($pIC_{50}$) was expressed with acceptable statistical significance in model-II:

Model-II

$$pIC_{50} = 2.32669 + 0.635201(\text{AlogP}) - 0.422558(\text{Atype\_C\_8}) + (\text{Atype\_C\_25}) + 0.181123(\text{Atype\_H\_52}) - 0.285327(\text{logP}) + 0.58861(\text{Chiral Centers}) + 0.4322249(\text{S\_dssC})$$

$N_{(\text{Training set})} = 35$, $r^2 = 0.863$, $F = 24.296$, $r = 0.929$, $r^2_{CV} = 0.775$, $r^2_{BS} = 0.864$, PRESS = 3.915, $N_{(\text{test set})} = 8$, $r^2_{pred} = 0.888$

where F is the value of ratio between regression and residual variances (Song *et al.*, 2008). The inter-correlation of the descriptors appeared in the above best model was taken into account and the descriptors were found to be reasonably orthogonal. Model-II contains much more significant descriptors than model-1. The high $r^2_{pred}$ value of 0.888 for the test set accounted for good predictive ability. According to model-II, it can explain and predict 86.3% and 88.8% of descriptors, respectively, which can be proved in predicting the test set (Table 2). The residuals of model-II are also much smaller than that of model-I (Table 2). Thus the binding activity ($pIC_{50}$) should be considered in terms of various descriptors in each molecule.

Compared with model-I, model-II have the same descriptors of Atype_C_25, Atype_H_52

and AlogP with positive coefficients and logP with negative coefficient. According to model-II, the C5a receptor binding activity ($pIC_{50}$) is also affected by the descriptors of Atype_C_8, Chiral Centers and S_dssC. The negative slope of Atype_C_8 represents that activity decreases with an increase in lipophilicity related to C_8 atom types for these molecules. The atom type C_8 (Ghose and Crippen, 1987) is C in $:CHR_2X$ where R represents any group linked through carbon and X represents any heteroatom (O, N, S, and halogens). Chiral Centers is the count of the number of chiral centers (R or S) present in a molecule. It is positively correlated with the binding activity, indicating the more Chiral Centers a molecule has, the high C5a receptor binding activity is. S_dssC is a descriptor of E-state-indices and represents the atomic type of Atomic-type =C< in aliphatic hydrocarbon, where S stands for the sum of the E-state values for a given atom type in a molecule, d means double bonds and s means single bond. The E-state indices (Hall and Kier, 1995) encode information about both the topological environment and the electronic interaction of an atom due to all other atoms in the molecule. Increasing presence of these features in a molecule contribute more towards binding activity.

#### Conclusions

On the basis of present study, it has been concluded that the described 2D-QSAR analysis contributes to the identification of important physiochemical parameters in explaining the variation in activity in a set of 43 molecules. The 2D-QSAR models derived by GFA method and Stepwise-MLR method have moderate internal and external predictivity, as shown by the values of $r^2_{CV}$ of 0.752 and 0.775, and $r^2_{pred}$ of 0.801 and 0.888, respectively, highlighting the importance of hydrophobicity of molecules. The statistical significance and robustness of the model has been confirmed by the full cross-validation tests and the randomization tests. Hence the model can be useful in the optimization of activity in this class of molecules, leading to further designing more novel orally available non-peptide C5a receptor antagonists.

### Abbreviations

C5a, Complement anaphylatoxin 5a; C5aR, Complement anaphylatoxin 5a receptor; CS, Complement system; ARDS, Adult respiratory distress syndrome; QSAR, Quantitative structure-activity relationship; GFA, Genetic function approximation; LOF, Friedman's lack of fit score; Stepwise-MLR, Stepwise multiple linear regression

#### Acknowledgements

Authors acknowledge Prof ZG Li for providing Cerius$^2$ (version 4.10) on Silicon Graphics O2

Nature Precedings : doi:10.1038/npre.2011.6630.1 : Posted 19 Nov 2011

R5000 workstation.

## Figures and Tables

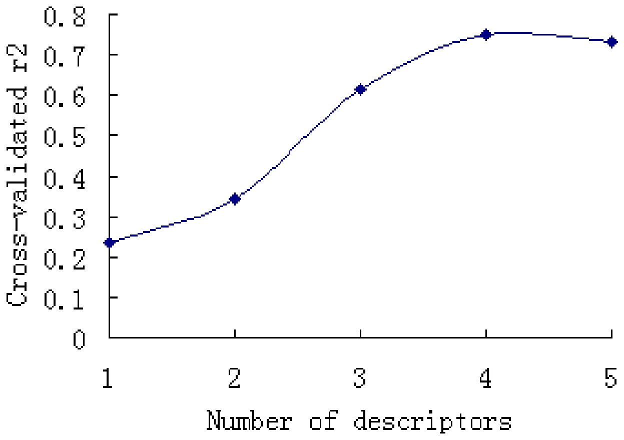

**Figure 1**. Plot of cross-validated $r^2$ ($r^2_{CV}$) as a function of the number of descriptors adequate in the final QSAR model

**Table 1.** Structures of non–peptide C5a receptor antagonists used in present QSAR study

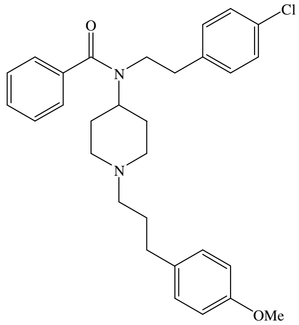

Compound **1**

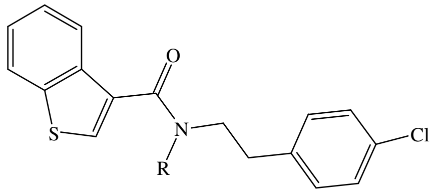

(a)

A series of piperidine analogues of compound **2**

| NO. | R | NO. | R |
|---|---|---|---|
| **2** |  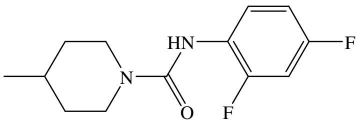 | **8** |  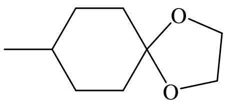 |
| **3** |  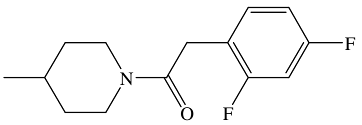 | **9** | Cyclohexyl |
| **4** |  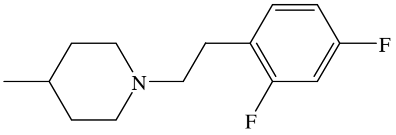 | **10** | Benzyl |
| **5** |  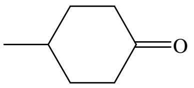 | **11** |  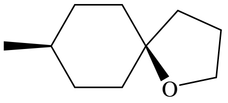 |
| **6** |  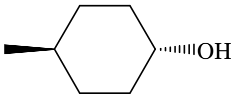 | **12** |  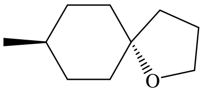 |
| **7** |  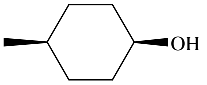 | | |

Nature Precedings : doi:10.1038/npre.2011.6630.1 : Posted 19 Nov 2011

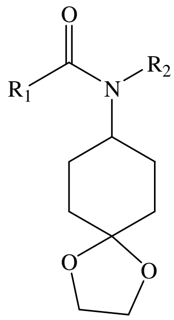

(b)

A series of benzothiophene analogues (**13-20**) and p-chloro-phenethylamine analogues (**21-28**) of compound **8**

| NO. | $R_1$ | $R_2$ | NO. | $R_1$ | $R_2$ |
|---|---|---|---|---|---|
| **13** |   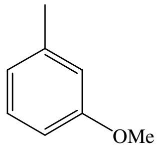 |   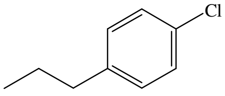 | **21** |   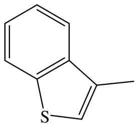 |   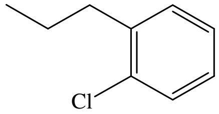 |
| **14** |  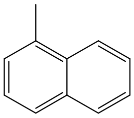 |   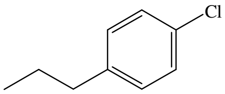 | **22** |   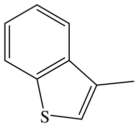 |   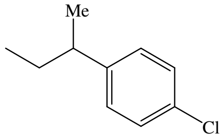 |
| **15** |   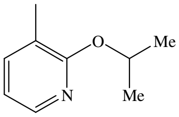 |   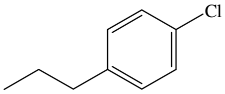 | **23** |   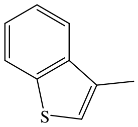 | **Benzyl** |
| **16** |   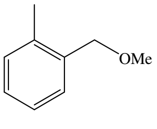 |   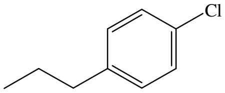 | **24** |   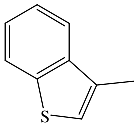 |   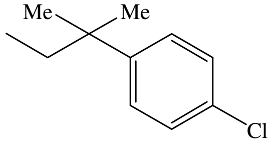 |
| **17** |   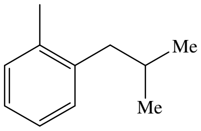 |   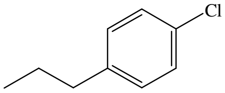 | **25** |   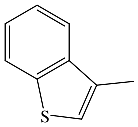 |   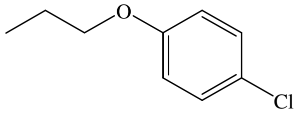 |
| **18** |   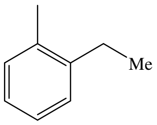 |   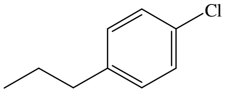 | **26** |   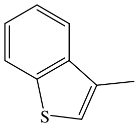 |   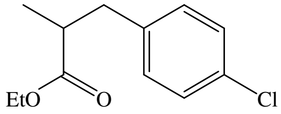 |
| **19** |   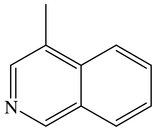 |   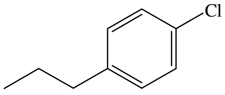 | **27** |   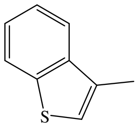 |   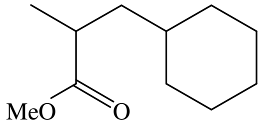 |

Nature Precedings : doi:10.1038/npre.2011.6630.1 : Posted 19 Nov 2011

Some non-peptide C5a receptor antagonists

**20**

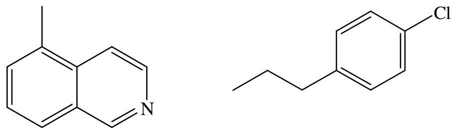

**28**

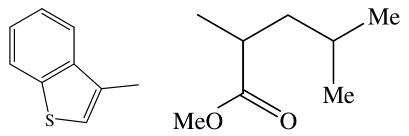

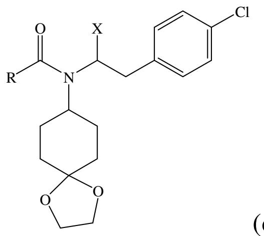

(c)

α-Amido analogues of compound **43**

| NO. | R | X | NO. | R | X |
|---|---|---|---|---|---|
| **29** | 1-Naphthyl | $CO_2Et$ | **34** | 3-Benzo-thiophene | C(O)Me |
| **30** | 3-Benzo-thiophene | $CO_2Me$ | **35** | 1-Naphthyl | $CH_2NMe_2$ |
| **31** | 2-Ethyl-Phenyl | $CO_2H$ | **36** | 2-Ethyl-Phenyl | $CH_2NMe_2$ |
| **32** | 2-Ethyl-Phenyl |  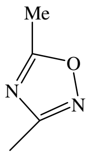 | **37** | 2-Ethyl-Phenyl |  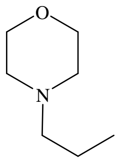 |
| **33** | 3-Benzo-thiophene | $CH_2OMe$ | **38** | 2-(2-Dimethylaminomethyl)-phenyl | H |

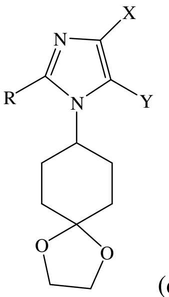

(d)

Some heterocyclic templates

Nature Precedings : doi:10.1038/npre.2011.6630.1 : Posted 19 Nov 2011

| NO. | R | X | Y |
|---|---|---|---|
| **39** | 1-Naphthyl | H | $CH_2$(p-Cl-Phenyl) |
| **40** | 3-Benzo-thiophene | H | $CH_2$(p-Cl-Phenyl) |
| **41** | 3-Benzo-thiophene | H | $CH_2$-cyclo-hexyl |
| **42** | 3-Benzo-thiophene | Me | $CH_2$(p-Cl-Phenyl) |

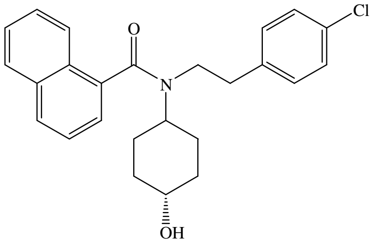

Compound **43**

**Table 2**. Actual, predicted activities and main descriptors of training set and test set molecules

| No. | [125]Binding affinity $IC_{50}$(nM) | $pIC_{50}$ (M) | Model-I[b] | | Model-II[c] | | AlogP | logP |
|---|---|---|---|---|---|---|---|---|
| | | | Predicted | Residual[d] | Predicted | Residual[d] | | |
| 1 | 1000 | 6.000 | 5.920 | 0.080 | 5.935 | 0.065 | 6.07 | 6.410 |
| 2[a] | 31 | 7.509 | 6.755 | 0.754 | 6.362 | 1.147 | 5.38 | 1.190 |
| 3 | 330 | 6.481 | 6.620 | -0.139 | 6.376 | 0.105 | 5.56 | 3.390 |
| 4 | 200 | 6.699 | 7.125 | -0.426 | 7.060 | -0.361 | 6.44 | 4.650 |
| 5 | 350 | 6.456 | 6.458 | -0.002 | 6.610 | -0.154 | 4.90 | 3.080 |
| 6 | 70 | 7.155 | 7.023 | 0.132 | 6.753 | 0.402 | 4.69 | 2.750 |
| 7 | 200 | 6.699 | 7.023 | -0.324 | 6.753 | -0.054 | 4.69 | 2.750 |
| 8 | 75 | 7.125 | 6.994 | 0.131 | 7.000 | 0.125 | 5.07 | 1.710 |
| 9 | 175 | 6.757 | 6.684 | 0.073 | 6.746 | 0.011 | 5.75 | 4.200 |
| 10 | 370 | 6.432 | 6.448 | -0.016 | 6.722 | -0.290 | 5.84 | 3.210 |
| 11 | 5 | 8.301 | 7.527 | 0.774 | 7.838 | 0.463 | 5.03 | 3.820 |
| 12 | 200 | 6.699 | 6.760 | -0.061 | 7.077 | -0.378 | 4.09 | 3.290 |

Nature Precedings : doi:10.1038/npre.2011.6630.1 : Posted 19 Nov 2011

| | | | | | | | | |
|---|---|---|---|---|---|---|---|---|
| 13 | 1000 | 6.000 | 5.814 | 0.186 | 5.836 | 0.164 | 4.58 | 3.130 |
| 14[a] | 80 | 7.097 | 7.227 | -0.130 | 7.238 | -0.141 | 5.83 | 3.960 |
| 15 | 835 | 6.078 | 6.819 | -0.714 | 6.557 | -0.479 | 4.72 | 3.120 |
| 16 | 175 | 6.757 | 6.335 | 0.422 | 6.355 | 0.402 | 4.57 | 2.770 |
| 17[a] | 1.5 | 8.824 | 6.803 | 2.021 | 6.846 | 1.978 | 6.42 | 5.280 |
| 18[a] | 18 | 7.745 | 6.601 | 1.144 | 6.646 | 1.099 | 5.69 | 4.350 |
| 19 | 400 | 6.398 | 6.731 | -0.333 | 6.735 | -0.337 | 4.52 | 2.710 |
| 20 | 110 | 6.959 | 6.762 | 0.197 | 6.778 | 0.181 | 5.07 | 1.530 |
| 21 | 300 | 6.523 | 7.049 | -0.526 | 7.044 | -0.521 | 5.47 | 2.110 |
| 22 | 35 | 7.456 | 6.990 | 0.466 | 7.548 | -0.092 | 4.20 | -0.070 |
| 23 | 395 | 6.403 | 6.245 | 0.158 | 6.170 | 0.233 | 5.98 | 2.730 |
| 24 | 90 | 7.046 | 6.988 | 0.058 | 6.919 | 0.127 | 4.66 | 1.280 |
| 25 | 435 | 6.362 | 6.134 | 0.228 | 6.065 | 0.297 | 4.92 | -0.640 |
| 26 | 5 | 8.301 | 7.764 | 0.537 | 8.112 | 0.189 | 4.51 | -0.900 |
| 27 | 89 | 7.051 | 7.009 | 0.042 | 6.942 | 0.109 | 3.83 | -2.150 |
| 28 | 290 | 6.538 | 6.934 | -0.396 | 6.825 | -0.287 | 3.83 | -2.150 |
| 29 | 3 | 8.523 | 8.476 | 0.047 | 8.398 | 0.125 | 5.94 | 1.610 |
| 30 | 15 | 7.824 | 8.246 | -0.422 | 8.027 | -0.203 | 5.75 | -0.880 |
| 32 | 350 | 6.456 | 6.858 | -0.402 | 6.450 | 0.006 | 5.43 | 2.940 |
| 32 | 8 | 8.097 | 8.130 | -0.033 | 8.191 | -0.094 | 7.44 | 3.180 |
| 33 | 12 | 7.921 | 7.654 | 0.267 | 7.762 | 0.159 | 5.89 | 1.350 |
| 34 | 45 | 7.347 | 8.107 | -0.760 | 8.112 | -0.765 | 6.27 | 0.700 |
| 35[a] | 13 | 7.886 | 7.423 | 0.463 | 7.586 | 0.300 | 5.88 | 3.430 |
| 36 | 27 | 7.569 | 6.797 | 0.772 | 6.993 | 0.576 | 5.74 | 3.720 |
| 37[a] | 200 | 6.699 | 6.704 | -0.005 | 6.911 | -0.212 | 5.45 | 3.490 |
| 38 | 500 | 6.301 | 6.395 | -0.094 | 6.434 | -0.133 | 4.72 | 2.890 |
| 39 | 90 | 7.046 | 7.055 | -0.009 | 6.961 | 0.085 | 6.53 | 5.060 |
| 40 | 500 | 6.301 | 7.074 | -0.773 | 6.954 | -0.653 | 6.45 | 3.480 |
| 41[a] | 47 | 7.328 | 6.361 | 0.967 | 6.264 | 1.064 | 6.12 | 3.750 |
| 42[a] | 90 | 7.046 | 7.055 | -0.009 | 6.938 | 0.108 | 6.62 | 3.915 |
| 43 | 125 | 6.903 | 7.226 | -0.323 | 6.964 | -0.061 | 5.45 | 5.100 |

[a] Molecules used in the test set.

[b] Model-I is the best model generated by GFA method

[c] Model-II is the best model generated by Stepwise-MLR method

[d] Residual = Actual $pIC_{50}$ - Predicted $pIC_{50}$

Nature Precedings : doi:10.1038/npre.2011.6630.1 : Posted 19 Nov 2011

**Table 3.** Descriptors used for building 2D-QSAR models

| Type | Descriptors |
|---|---|
| E-state-indices | Electrotopological-state indices |
| Spatial | Radius of gyration, principal moment of inertia, shadow indices molecular surface area, density, molecular volume, molecular area |
| Electronic | Sum of atomic polarizabilities, dipole moment, energy of highest occupied orbital (HOMO), energy of lowest unoccupied orbital (LUMO), superdelocalizability |
| Thermodynamic | Ghose and Crippen molar refractivity, heat of formation, log of the partition coefficient, log of the partition coefficient atom type value, desolvation free energy for water, desolvation free energy for octanol |
| Structural | Number of chiral centers, number of rotatable bonds, number of hydrogen-bond donors, number of hydrogen-bond acceptors, molecular weight |
| Conformational | The energy of the currently selected conformation |
| Information_content | Multigraph information content indices, information of atomic composition index |
| Topological | Kier and Hall molecular connectivity index, Hosoya index, molecular flexibility index, Balaban indices, Zagreb index, Logarithm of Hosoya index |

**Table 4.** Results of full cross-validation tests for 2D-QSAR models generated by GFA method

| Rule | PRESS | Sum of sq dev. | $r^2_{CV}$ |
|---|---|---|---|
| Leave-1-out | 5.442 | 2.135 | 0.752 |
| Leave-2-out | 5.421 | 2.135 | 0.754 |
| Leave-3-out | 5.615 | 2.135 | 0.726 |
| Leave-4-out | 5.513 | 2.135 | 0.736 |
| Leave-5-out | 5.694 | 2.135 | 0.764 |
| Leave-6-out | 5.236 | 2.135 | 0.746 |
| Leave-7-out | 5.789 | 2.135 | 0.750 |
| Leave-8-out | 5.134 | 2.135 | 0.761 |
| Leave-9-out | 5.394 | 2.135 | 0.746 |
| Leave-10-out | 5.264 | 2.135 | 0.765 |

Nature Precedings : doi:10.1038/npre.2011.6630.1 : Posted 19 Nov 2011

**Table 5.** Results of randomization tests for 2D-QSAR models generated by GFA method

| Randomization test: | | | | |
|---|---|---|---|---|
| Confidence level | 90% | 95% | 98% | 99% |
| Total trials | 9 | 19 | 49 | 99 |
| r from non-random | 0.879 | 0.879 | 0.879 | 0.879 |
| Random r′s< non-random | 9 | 19 | 49 | 99 |
| Random r′s> non-random | 0 | 0 | 0 | 0 |
| Mean of r from random trial | 0.550 | 0.534 | 0.587 | 0.512 |
| Standard deviation of random trials | 0.051 | 0.059 | 0.067 | 0.045 |
| Standard deviation from non-random r to mean | 4.263 | 4.169 | 4.255 | 4.124 |

**Table 6.** Frequency distribution of the variables

| Descriptor | Frequency |
|---|---|
| AlogP | 86 |
| logP | 75 |
| Atype_H_52 | 46 |
| Atype_C_25 | 25 |
| Chiral Centers | 19 |
| S_dssC | 15 |
| S_aaCH | 11 |
| Atype_O_59 | 9 |

Nature Precedings : doi:10.1038/npre.2011.6630.1 : Posted 19 Nov 2011
